# Potential target within the tumor microenvironment - MT1-MMP

**DOI:** 10.3389/fimmu.2025.1517519

**Published:** 2025-03-24

**Authors:** Jinlong Liu, Yijing Li, Xueqi Lian, Chenglin Zhang, Jianing Feng, Hongfei Tao, Zhimin Wang

**Affiliations:** ^1^ School of Basic Medical Sciences, Zhengzhou University, Zhengzhou, China; ^2^ Centre for Cell and Gene Therapy, Academy of Medical Sciences, Zhengzhou University, Zhengzhou, China

**Keywords:** MT1-MMP_1_, tumor microenvironment_2_, targeted therapy_3_, extracellular matrix_4_, metastasis_5_

## Abstract

Matrix metalloproteinases are integral to the modification of the tumor microenvironment and facilitate tumor progression by degrading the extracellular matrix, releasing cytokines, and influencing the recruitment of immune cells. Among the matrix metalloproteinases, membrane-type matrix metalloproteinase 1 (MT1-MMP/MMP14) is the first identified membrane-type MMP and acts as an essential proteolytic enzyme that enables tumor infiltration and metastatic progression. Given the pivotal role of MT1-MMP in tumor progression and the correlation between its overexpression in tumors and unfavorable prognoses across multiple cancer types, a comprehensive understanding of the potential functional mechanisms of MT1-MMP is essential. This knowledge will aid in the advancement of diverse anti-tumor therapies aimed at targeting MT1-MMP. Although contemporary research has highlighted the considerable potential of MT1-MMP in targeted cancer therapy, studies pertaining to its application in cell therapy remain relatively limited. In this review, we delineate the structural characteristics and regulatory mechanisms of MT1-MMP expression, as well as its biological significance in tumorigenesis. Finally, we discussed the current status and prospects of anti-tumor therapies targeting MT1-MMP.

## 1 Introduction

**Figure 1:**
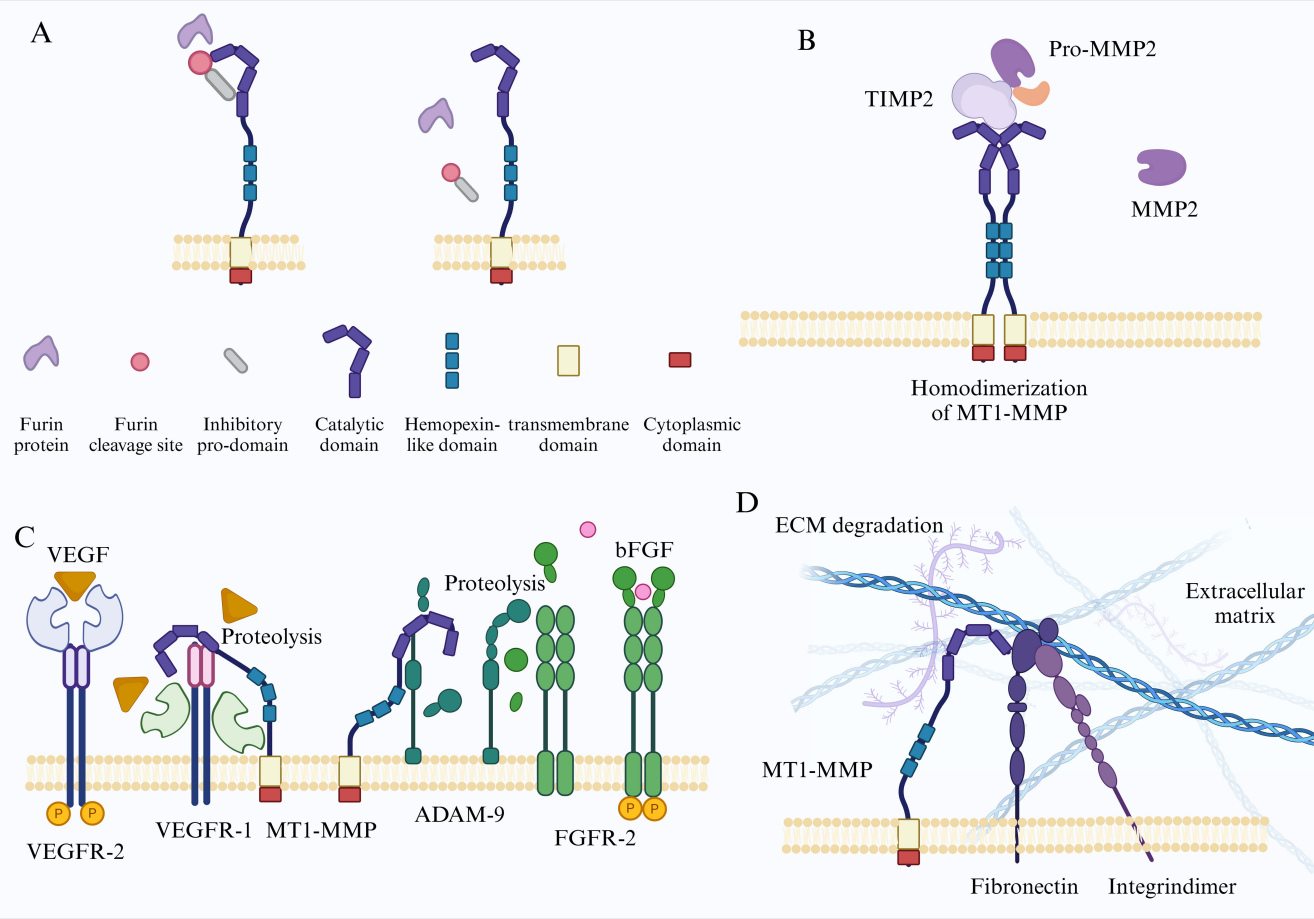
**(A)** Illustrates the structural diagram of the inactive MT1-MMP, with distinct colors denoting various functional segments. MT1-MMP is subjected to hydrolysis by the Furin protein within the trans-Golgi apparatus, resulting in the release of the catalytic structural domain through the removal of the pro-structural domain. **(B)** Following the dimerization of the hemagglutinin structural domain at the cell membrane, MT1-MMP associates with the proMMP2-TIMP2 dimer, leading to the removal of the proMMP2 pre-structural domain and the subsequent release of its catalytic structural domain. **(C)** MT1-MMP facilitates angiogenesis through the regulation of VEGF and bFGF signaling pathways. **(D)** MT1-MMP is involved in collagen hydrolysis and the degradation of the extracellular matrix, thereby promoting tumor metastasis.

According to the most recent publication from the International Agency for Research on Cancer of the World Health Organization, cancer represents a significant threat to global public health (1). Matrix metalloproteinases (MMPs) constitute a class of metal ion-dependent proteases that are responsible for the degradation of extracellular matrix (ECM) and basement membrane components. These enzymes possess highly structurally similar active sites within their catalytic domains and share a conserved domain (2). MMPs are crucial in various biological processes, including morphogenesis, wound healing, tissue remodeling, and repair, as well as in the progression of numerous diseases, particularly cancer (3, 4). It is important to note that MT1-MMP is overexpressed in pancreatic cancer, non-small cell lung cancer, breast cancer, and various other cancer types, as well as in their microenvironments (5–7). (**Figure 1A**). The overexpression of MT1-MMP is frequently correlated with enhanced tumor invasiveness, increased metastatic potential, treatment resistance, and poor patient prognosis (8, 9). This review aims to summarize the structural characteristics and regulatory mechanisms governing the expression of MT1-MMP, examine its physiological roles in tumorigenesis and development, and discuss the current advancements in antitumor therapies targeting MT1-MMP. This analysis will assist in identifying gaps in existing research and in the development of targeted anti-tumor therapies. Finally, we will explore the potential and limitations of MT1-MMP as a novel target for anti-tumor interventions.

## 2 The fundamental structure and functional regulation of MT1-MMP

### 2.1 Structure of MT1-MMP

Since the identification of the first member of the MMPs family, MMP1, in 1962, a total of 23 distinct types of human MMPs have been recognized (10). These enzymes are categorized into six groups based on their substrate specificity and structural characteristics: collagenases, gelatinases, stromelysins, matrilysins, membrane-type MMPs, and other MMPs (11). Typical MMPs are generally composed of an inhibitory pro-domain, a catalytic domain, a hinge region, and a hemopexin-like domain. Membrane-type MMPs (MT-MMPs) are characterized by the presence of a transmembrane domain and a cytoplasmic domain (11).

As a member of MT-MMPs, the gene encoding human MT1-MMP contains 582 amino acids, including a signal peptide (Met_1_-Thr_20_), an inhibitory pro-domain (Ala_21_-Arg_111_), a Furin protein cleavage site (Arg_108_-Ile_114_), a catalytic domain (Tyr_112_-Ser_287_), a hinge region (Gly_288_-Gly_315_), a hemopexin-like domain (Pro_316_-Gly_507_), Linker (Cys_508_-Ala_541_), a transmembrane domain (Val_542_Phe_562_), and a cytoplasmic domain (Arg_563_-Val_582_) (**Figure 1A**) (12). The pro-domain masks the catalytic domain to inhibit its proteolytic function, the Furin cleavage site is involved in regulating the activation process of MT1-MMP, hemopexin-like domain is used for dimerization substrate binding and phospholipid bilayer interaction (13), and transmembrane domain allows MT1-MMP to anchor to the cell membrane surface. The cytoplasmic domain, consisting of only 20 amino acids, contains multiple post-translational modified amino acids, serving as a hub for signal transduction and participating in a series of physiological processes such as the transport localization, activation, and cell adhesion of MT1-MMP (14).

### 2.2 Regulation of MT1-MMP gene expression

#### 2.2.1 The interaction between cis-acting elements and trans-acting factors regulates gene expression

**Figure 2:**
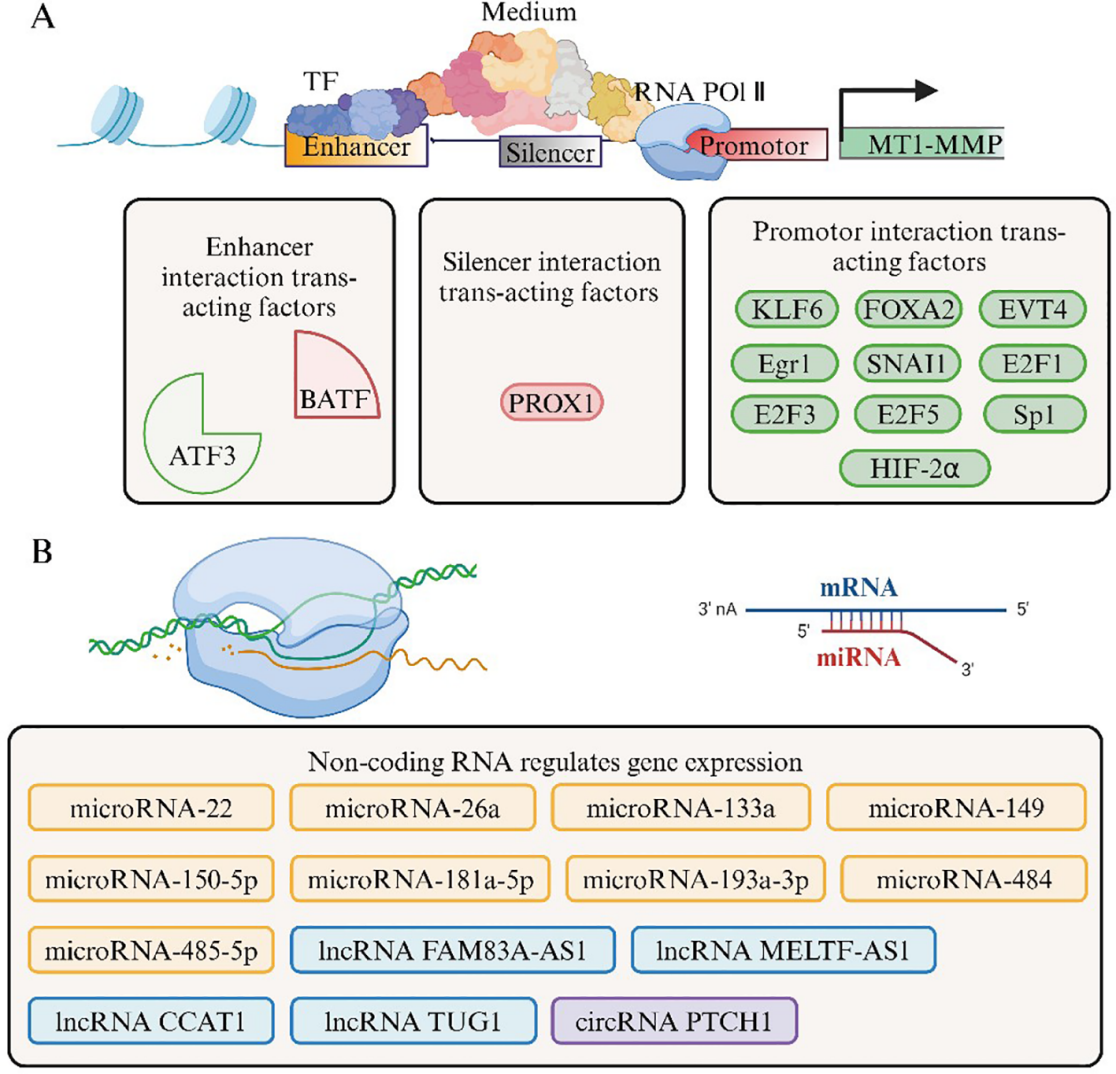
Regulation of MT1-MMP Gene Expression. **(A)** The transcription process of the MT1MMP gene involves interactions between various cis-acting elements and trans-acting factors. In this diagram, green indicates promotion of gene expression, while red signifies inhibition. **(B)** Posttranscriptional mRNA levels illustrate the regulation of MT1-MMP gene expression by various noncoding RNAs. Among these, microRNAs act directly, whereas lncRNAs and circRNAs exert regulatory functions by modulating microRNAs.

As a member of MT-MMPs family, MT1-MMP plays a crucial role in the maintenance of ECM homeostasis. Research has shown that MT1-MMP is integral to the remodeling of the extracellular matrix, with its aberrant regulation being strongly linked to multiple pathological phenomena (15). Consequently, it is subject to multi-layered regulatory mechanisms. At the transcriptional level, the regulation of gene expression for MT1-MMP can be summarized as follows (**Figure 2A**). A comprehensive characterization of the promoter indicates that MT1-MMP is devoid of a TATA box, possesses a distinctive binding site for the Sp1 transcription factor, features multiple transcription start sites, and includes an upstream repressive regulatory element (16). Research has demonstrated that the infection of lymphatic endothelial cells (LECs) by Kaposi sarcoma herpesvirus (KSHV) results in the downregulation of PROX1 expression, which is accompanied by the upregulation of MT1-MMP expression and the acquisition of an MT1-MMP-dependent invasive phenotype (17). Further studies indicate the presence of a PROX1 binding site located between 1139 and 1123 base pairs (bp) upstream of the transcription start site (TSS) of the MT1-MMP gene. Additionally, four consecutive PROX1 binding sites are identified between 1020 and 963 bp upstream of the TSS, with PROX1 binding capable of inhibiting the expression of downstream genes. The absence of PROX1 expression can promote the upregulation of MMP14 expression in tumor cells, while the reestablished expression of PROX1 inhibits MT1-MMP expression and suppresses the three-dimensional sprouting and invasion of cancer cells. Moreover, the increased levels of MT1-MMP in the dermal lymphatics of mice suggest that PROX1 suppresses MT1-MMP expression under physiological conditions (18). It is crucial to emphasize that MT1-MMP is currently the only known inhibitory target of PROX1, making PROX1 the first identified direct inhibitor of MT1-MMP (18). The overexpression of MT1-MMP in gastric cancer has been confirmed to be associated with a favorable prognosis for patients with gastric cancer (19). In intestinal tumors characterized by PROX1 overexpression and defective membrane types, PROX1 plays a crucial role in tumor stroma activation and modulates the sensitivity of tumors to chemotherapy in an MT1-MMP-dependent manner (20).

During endothelial tissue injury, the transcription factor Krüppel-like factor 6 translocates to the nucleus, where it interacts with the promoter of MT1-MMP and enhances the transcriptional activity of MT1-MMP (21). Notably, in fibro-adipogenic progenitor cells, microRNA-22-3p targets the KLF6/MT1-MMP axis, thereby regulating fat infiltration during muscle degeneration (22). In renal cell carcinoma, hypoxia-inducible factor 2 alpha functions synergistically with specificity protein 1 within the promoter region of MT1-MMP, thereby enhancing the expression of MT1-MMP and contributing to increased invasiveness (23). In primary breast cancer, N-α-acetyltransferase D is typically upregulated and is responsible for mediating the N-a-terminal acetylation of histone H4. The absence of NatD expression results in a diminished accumulation of N-terminal acetylated histone H4 at the promoter region of FOXA2, which directly inhibits the expression of FOXA2 and consequently suppresses the expression of MT1-MMP (24). A study investigating angiogenesis in liver cancer demonstrates that EVT4 regulates the expression of MT1-MMP at the transcriptional level, thereby promoting angiogenesis, migration, and invasion within the hepatocellular carcinoma microenvironment (25). In a separate investigation, E2F1, E2F3, and E2F5 have been identified as key factors in the transcriptional regulation of the MT1-MMP gene. Notably, E2F1 plays a critical role in modulating MT1-MMP expression, which is tightly regulated by the retinoblastoma protein. This finding was corroborated in tumor cells derived from human papillomavirus, where the HPV derived E7 oncoprotein facilitates the degradation of the Rb protein. This process subsequently activates E2F and leads to the upregulation of MT1-MMP expression (26–28). Furthermore, within the tumor microenvironment, physical stimulation enhances the expression levels of transcription factors and early growth response protein 1, consequently facilitating the upregulation of MT1-MMP (29). However, it is important to note that even the same transcription factors can have different regulatory roles in various tissues. Studies have demonstrated that the transcription factors E2F1 and E2F3 are expressed in the placenta but do not play a role in the regulatory control of MT1-MMP expression (30).

**Figure 3:**
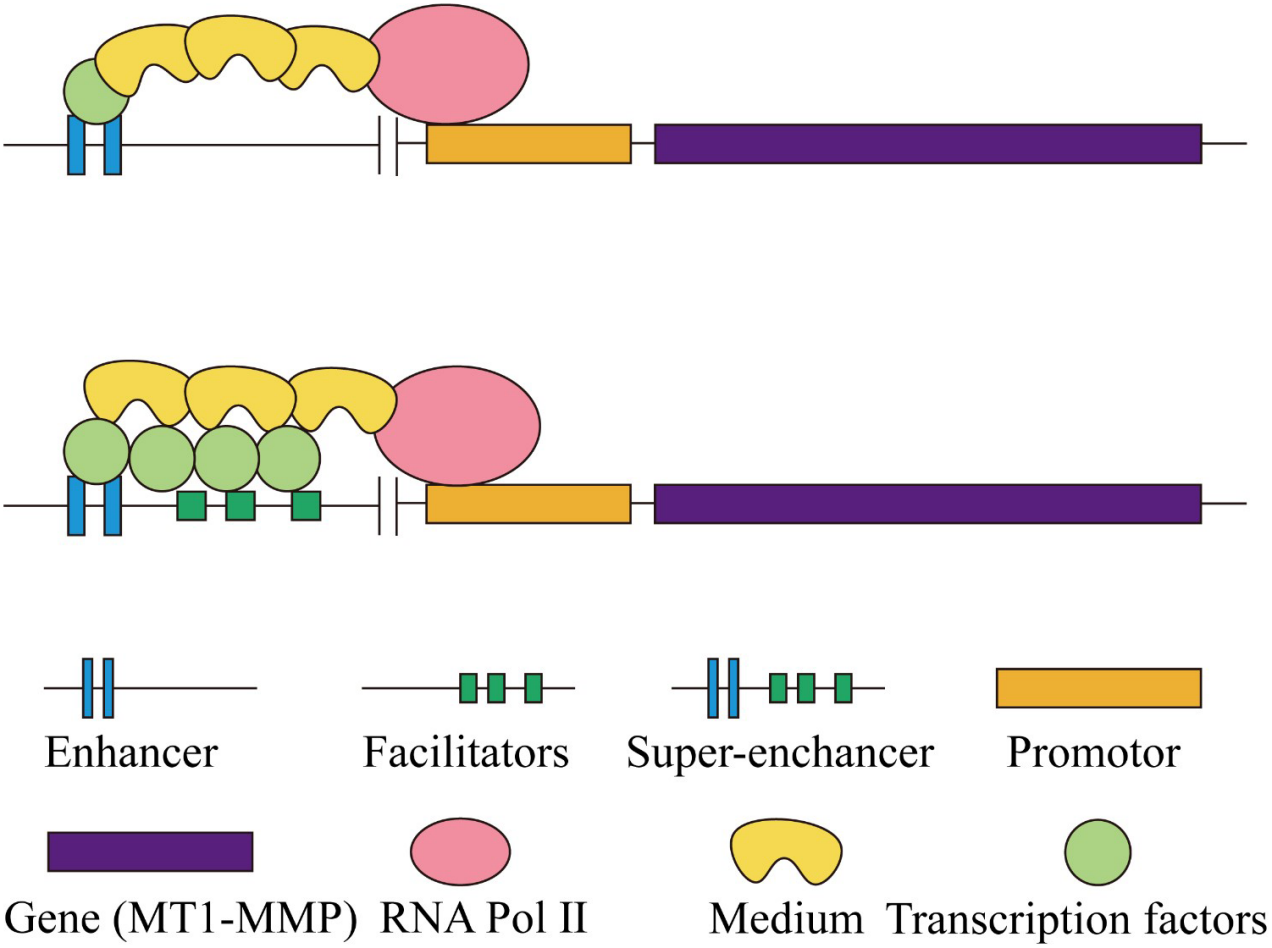
A schematic diagram illustrating the differences in gene expression regulation between classic enhancers and super-enhancers.

Super-enhancers (SEs) are defined as genomic regions that contain multiple closely spaced enhancers, distinguished by a high concentration of transcription factors, co-factors, and epigenetic modifications (31). However, Joseph W. Blayney and others argue that SEs contain classic enhancers and promoters, where the promoters do not possess inherent enhancer activity but can enhance the function of traditional enhancers (32) (**Figure 3**). Recent studies suggest that specific signaling events within tumors function as critical oncogenic drivers that facilitate tumorigenesis. Furthermore, the localization of these SEs indicates the overactivation of numerous proto-oncogenes (33). Recent research suggests that in tongue squamous cell carcinoma, the transcription factors BATF and ATF3 interact with super-enhancer regions in a switch-like manner to collaboratively regulate the expression of MT1-MMP. Among them, ATF3 facilitates the transcription of MT1-MMP by engaging with the enhancer region, whereas BATF inhibits this interaction, resulting in a reduction of MT1-MMP expression (34).

It is noteworthy that, similar to the regulation of promoter methylation in most human genes, the promoter region of MT1-MMP encompasses a CpG island. Methylation of this promoter region serves to inhibit both gene expression and cell migration (35, 36). This observation aligns with the minimal levels of methylation observed in the MT1-MMP promoter within highly invasive glioma cells, in contrast to the elevated levels of methylation found in the MT1-MMP promoter of noninvasive breast cancer cell lines (28, 37, 38).

#### 2.2.2 Non-coding RNA regulates gene expression

MicroRNA, circular RNA (circRNA), and long non-coding RNA (lncRNA) are all constituents of the non-coding RNA category and have emerged as significant focal points in recent research concerning the regulation of non-coding gene expression. The regulatory mechanisms of non-coding RNAs that target MT1-MMP have been identified in various pathological processes (**Figure 2B**). In the context of rheumatoid arthritis, microRNA-150-5p, which is derived from mesenchymal stem cells (MSCs), targets MT1-MMP and vascular endothelial growth factor. This targeting results in a reduction of the migration and invasion of fibroblast-like synoviocytes associated with rheumatoid arthritis (39). In lung adenocarcinoma, lncRNA FAM83A-AS1 facilitates the proliferation, invasion, and epithelialmesenchymal transition (EMT) of lung adenocarcinoma cells by targeting MT1-MMP via microRNA-150-5p (40). CircRNA PTCH1 facilitates the invasion and metastasis of renal cell carcinoma via the microRNA-485-5p/MT1-MMP axis and the EMT process (41). In osteosarcoma, RREB1 transcriptionally enhances the expression of the lncRNA MELTF-AS1, which in turn regulates the expression of MT1-MMP through its interaction with microRNA-485-5p (42). MicroRNA-26a exerts its effects by targeting MT1-MMP and matrix metalloproteinase 16, thereby inhibiting the proliferation, migration, and invasion of skin squamous cell carcinoma (43). In the context of colorectal cancer, microRNA-26a-5p is influenced by the regulation of lncRNA TUG1, thereby facilitating the progression of colorectal cancer via the MT1-MMP/p38 MAPK/Hsp27 signaling pathway (44). MicroRNA-22 targets MT1-MMP and SNAI1 to inhibit the growth and metastasis of gastric cancer, with SNAI1 serving to induce the expression of MT1-MMP (45). In fibroblast progenitor cells, microRNA-22-3p plays a role in the regulation of fat infiltration during muscle degeneration (22). Additionally, microRNA-193a-3p, microRNA-133a, and microRNA-149 have been identified as regulators of MT1-MMP, significantly influencing the proliferation, migration, and invasion of cells related to intervertebral disc herniation, osteosarcoma, and pituitary adenoma, respectively (46–48). In the context of cervical cancer, decreased CpG methylation of the microRNA-484 promoter, which is reliant on the activity of DNA methyltransferase DNMT1, enhances the expression of microRNA-484. This upregulation subsequently inhibits the expression of MT1-MMP and hepatocyte nuclear factor 1A, thereby exerting a negative regulatory effect on the WNT/MAPK and TNF signaling pathways (49). Furthermore, lncRNA CCAT1 is significantly overexpressed in cervical cancer and facilitates the proliferation and invasion of cancer cells via the microRNA-181a-5p/MT1-MMP signaling pathway (50).

### 2.3 Regulation of MT1-MMP protein activity

The function of MT1-MMP is subject to stringent regulation not only at the gene expression level but also at various levels concerning its protein activity. In this section, we examine the regulatory mechanisms governing MT1-MMP activity across different cellular regions. While the complete elucidation of these regulatory mechanisms remains incomplete, it is clear that they are essential for the accurate regulation of MT1-MMP protein activity, which is essential for maintaining cellular homeostasis mediated by MT1-MMP.

#### 2.3.1 Activity regulation based on protein hydrolysis and interactions

Similar to other members of MT-MMPs family, MT1-MMP is initially synthesized as an inactive precursor that includes a pro-domain within the cytoplasm (51). Following this, in the trans-Golgi network, MT1-MMP undergoes activation via furin and associated proprotein convertases through a hydrolytic process that cleaves the pro-domain, thereby releasing the catalytic domain (52) (**Figure 1A**). In LoVo cells derived from colorectal cancer, proMT1-MMP is translocated to the cell membrane in a manner that is resistant to Brefeldin A. This process is followed by autocatalytic activation, suggesting that the activation of its catalytic activity can occur through multiple mechanisms (53). Furthermore, active MT1-MMP possesses the ability to self-catalyze the generation of inactive fragments, as well as the release of its entire catalytic domain at the cell surface. Notably, investigations into the activity and sequence of both self-catalytic and non-self-catalytic shed fragments have demonstrated that the fragments resulting from the self-catalysis of MT1-MMP are situated within the catalytic domain of MMPs. This finding suggests that the shedding of the extracellular domain plays a crucial role in modulating MT1-MMP activity, maintaining a delicate equilibrium between active and inactive enzyme-solubilized fragments (54, 55). Another study indicates that the inactive membrane form 44-kDa product (44-MT1) generated by self-catalysis of MT1-MMP enhances the level of active enzyme on the cell surface by delaying the endocytosis rate of the 55-kDa active enzyme (56, 57), although the regulatory mechanism has not yet been completely clarified.

While MT1-MMP is capable of anchoring to the cell surface in its activated state, the manifestation of certain functions is contingent upon the homodimerization of MT1-MMP. When collagen serves as a substrate, its degradation process depends on the homodimerization of its hemopexin-like domain (58). The activation of pro-MMP2, which is reliant on MT1-MMP and TIMP2, necessitates the proximity of an additional free MT1-MMP to the trimeric complex. This proximity facilitates the cleavage of the pro-peptide of pro-MMP2, a process that is accomplished through the formation of a homodimeric complex of MT1-MMP (**Figure 1B**). Disrupting the formation of this dimer can effectively inhibit the activation of proMMP-2 on the cell surface (59).

Endogenous tissue inhibitors of metalloproteinases (TIMPs) constitute a class of proteins characterized by molecular weights between 21 and 28 kDa, which possess the ability to reversibly associate with MMPs, thereby modulating their activity (60). Despite the high structural similarity and substrate compatibility among TIMPs, their expression demonstrates notable tissue specificity and certain selectivity (61). To date, only four types of TIMPs have been found in humans, with the other three showing inhibitory functions targeting MT1-MMP, aside from TIMP-1 (62). It is worth mentioning that although TIMP2 is an inhibitor of MT1-MMP, during the hydrolysis of pro-MMP2 by MT1-MMP, a complex is formed between MT1-MMP and TIMP-2, with TIMP-2 acting as a receptor for pro-MMP2, forming a three-molecule complex that activates MMP2 (63). However, the TIMP2-dependent activation of pro-MMP2 by MT1-MMP does not seem to be unique, as evidenced in the MT1-MMP-dependent activation process of pro-MMP2 induced by tight junction protein claudins (64).

#### 2.3.2 Active regulation based on protein transport

Unlike secreted MMPs, the proteolytic activity of MT1-MMP is related to its expression level on the cell surface. After being synthesized in the ribosome and modified in the endoplasmic reticulum and Golgi apparatus, MT1-MMP is translocated to the cell membrane through the active exocytosis of Rab8-positive vesicles, a process facilitated by cytoplasmic microtubules and motor proteins (65). In the context of metastatic breast cancer, the inhibition of mammalian diaphanous-related formin 1 has been shown to impede the microtubule-mediated localization of MT1-MMP. This inhibition results in a diminished presence of active MT1-MMP on the membrane surface, thereby leading to a reduction in the invasive capabilities of breast cancer cells (66). Additionally, research has demonstrated that a complex formed between a subtype of plectin, referred to as iplectin (i=invadopodial), and MT1-MMP is instrumental in modulating the concentrations of cell surface enzymes through the initiation of invasive podosome formation (67).

Numerous studies have demonstrated that the effective internalization of MT1-MMP, mediated by clathrin and dynein, along with its transport through various endosomal compartments, serves a significant function in the coordinated regulation of the activity of cell surface MT1-MMP (68). Remacle et al. conducted co-localization studies utilizing various markers within endosomal compartments, revealing that a portion of internalized MT1-MMP can be recycled and subsequently relocated to the cell membrane surface. This recycling process is intricately associated with the targeted polarization of cellular invasive structures (69, 70). Rab GTPases serve as pivotal regulators of membrane transport pathways within cellular systems. Distinct isoforms of Rab GTPases exhibit co-localization in diverse endosomal compartments, thereby modulating the motility of each respective compartment. This regulatory pattern has been established to be contingent upon environmental factors (71, 72). Numerous studies have demonstrated that the processes of endocytosis and recycling of MT1-MMP can occur in a manner that is dependent on Rab GTPases (71, 73, 74). Furthermore, the transport and motility of MT1-MMP are modulated by various regulatory markers, including Rho GTPases, cdc42, RhoA, and Arf6, among others (75, 76).

While MT1-MMP is capable of internalizing from both early and recycling endosomes to late endosomes and subsequently recycling to the plasma membrane, a considerable proportion of MT1MMP undergoes degradation within mature lysosomes. Nevertheless, pertinent research has indicated that active MT1-MMP is released in exosomes during the cultivation of human fibrosarcoma (HT1080) and melanoma (G361) cell lines (77). Recent studies have demonstrated that the active form of MT1-MMP, which is secreted through exosomes, contributes to various pathological processes, including tumor cell invasion, the induction of an immune suppressive microenvironment, and tumor angiogenesis (78–80).

## 3 Activity of MT1-MMP and its biological functions in tumors

MT1-MMP, recognized as the first identified membrane-type matrix metalloproteinase (MT-MMP), has its discovery intricately linked to its role in the invasion of tumor cells (12). Among MMPs, MT1-MMP is the sole collagenolytic membrane-type MMP. This characteristic imparts distinct proteolytic functions to MT1-MMP, thereby solidifying its central role in the overall functionality of MMPs (81). Furthermore, in addition to its proteolytic function, the interaction of its transmembrane structure with the cytoskeleton enhances the roles of intercellular communication and intracellular signaling. Studies have shown that tumor cells with elevated levels of MT1-MMP exhibit an upregulation of hypoxia-inducible factor 1-alpha expression. This increase mediates the Warburg effect by inhibiting the activity of the hypoxia-inducible factor 1-alpha inhibitory factor through its cytoplasmic tail. Furthermore, the Ras-Raf-MEK1/2 signaling pathway is contingent upon the presence of MT1-MMP in response to growth factor stimuli. Moreover, MT1-MMP engages with CD44, subsequently promoting increased cellular motility by activating the epidermal growth factor receptor-mediated MAPK and PI3K signaling pathways. The heightened expression of MT1-MMP within neoplastic cells has been demonstrated to facilitate tumor-associated angiogenesis. Moreover, the formation of lamellipodia and the motility of myeloid progenitor cells have been observed in this context (82–85).

### 3.1 Tumor angiogenesis

During tumor development, new blood vessels are formed to ensure nutrient and oxygen supply within the tumor and provide a conducive environment for tumor metastasis. Research has shown that MT1-MMP participates in tumor angiogenesis by specifically upregulating the expression of VEGF-A through the activation of the Src tyrosine kinase signaling pathway (83). There are two receptors for VEGF on the cell membrane, namely, VEGFR-1 and VEGFR-2, with VEGFR-2 being the functional receptor and VEGFR-1 serving as a competitive inhibitory receptor with higher affinity (86). MT1-MMP can promote angiogenesis by cleaving VEGFR-1 to facilitate the binding of VEGF to VEGFR-2 (87, 88). Fibroblast growth factor 2 plays a crucial role in vascular development, and MT1-MMP facilitates FGF-2 signaling by hydrolyzing the metalloproteinase ADAM-9 and disintegrins, thereby promoting the process of vascular development (88, 89) (**Figure 1C**). In the context of pituitary adenomas, the pituitary tumor-transforming gene is significantly associated with tumor invasion. This correlation may be attributed to the elevated expression of PTTG, which appears to facilitate the upregulation of MT1-MMP, thereby inducing the expression of VEGF and ultimately contributing to the process of angiogenesis (90). In GBM, myeloid cells that express MT1MMP facilitate angiogenesis through various signaling pathways, including Visfatin, VEGF, and transforming growth factor beta (91). In ovarian cancer, knocking out the COG3 gene inhibits the expression of MT1-MMP, leading to suppressed angiogenesis (92). Additionally, in mouse models with constitutive endothelial cell-specific deletion of MT1-MMP, the growth and metastasis of melanoma are reduced, resulting in decreased vascular permeability (93). Notably, MT1-MMP may also play a role in inhibiting tumor progression. For example, the shedding of endoglin mediated by MT1-MMP leads to the generation of soluble endoglin, which in turn exerts an inhibitory effect on angiogenesis (94).

### 3.2 Tumor invasion and metastasis

#### 3.2.1 MT1-MMP in EMT

During tumorigenesis and development, MT1-MMP induces malignant transformation of various cancers under the pressure of tumor suppressor gene or oncogene mutations and the tumor microenvironment (95). Among the many tumor-associated malignant transformations, EMT is an important step in the process of tumor invasion and metastasis. Research has demonstrated that the presence of MT1-MMP within the tumor stroma can facilitate the invasion of cancer cells *in vivo*. This suggests that, beyond its role in inducing ECM degradation, MT1-MMP may also enhance tumor invasion through additional mechanisms. Research indicates that the inhibition of MT1-MMP can revert mesenchymal-like cancer cells, which express endogenous MT1-MMP, to a normal phenotype. This finding suggests that MT1-MMP within the tumor stroma may facilitate cancer cell invasion by promoting EMT. Furthermore, exogenous MT1-MMP has the capacity to induce EMT in adjacent cells that do not express MT1-MMP by elevating the levels of active TGF-β (96). This implies that although MT1-MMP is primarily expressed in stromal cells in most cancer tissues (97), cancer cell invasion can still be triggered by MT1-MMP produced through paracrine pathways from stromal cells.

#### 3.2.2 MT1-MMP in ECM degradation

During tumor invasion, overcoming the physical barrier of the ECM is necessary, and ECM degradation is closely related to MMPs (**Figure 1D**). Among them, MT1-MMP is a key enzyme for tumor cell invasion. As previously noted, MT1-MMP can accumulate at invadopodia via intracellular recycling. Invadopodia are specialized membrane protrusions that facilitate ECM degradation by invasive cells. Notably, a dual role of collagenolytic invadopodia was observed during cancer cell invasion (98). The ECM is mainly composed of collagen, fibronectin, laminin, and other components (88). Among all MT-MMPs, only MT1-MMP can cleave the glycine-leucine covalent bond in collagen, converting it into gelatin for further degradation (99). Furthermore, the degradation and renewal of fibronectin are crucial for tumor metastasis, morphogenesis, and motility (88). Research has demonstrated that the acidic tumor microenvironment promotes cancer cell motility mediated by MT1-MMP through the integrin β1/cofilin/F-actin signaling pathway (100). Under conditions of starvation, cancer cells exhibit a remarkable ability to sustain survival and proliferation by augmenting the degradation of the ECM through MT1-MMP, a process that is initiated by the mechanistic target of rapamycin in response to the nutrient-deprived environment (101). Furthermore, the stiffness of the ECM is associated with the invasiveness and EMT of different cancer cell types. Additionally, sensors that assess the mechanical characteristics of the ECM, including integrins, play a crucial role in facilitating MT1-MMP-mediated cell invasion within three-dimensional microenvironments (102). In cancer cells, alterations in the ECM remodeling capacity mediated by MT1-MMP may lead to alterations in the mechanical characteristics of the cellular microenvironment. These modifications possess the capacity to impact the EMT process (103),which may subsequently induce indirect variations in MT1-MMP expression (104).

#### 3.2.3 Ameboid-like invasion and MT1-MMP

Previously, we discussed how MT1-MMP mediates cancer cell invasion and metastasis through ECM degradation. Moreover, the processes through which cells invade are intricately linked to the structural and mechanical properties of the stroma, in addition to the ability of cells to modify the extracellular matrix. It is generally believed that cancer cells overcome ECM migration through a protease-dependent manner, while in cases where the stroma has larger pore sizes and mechanical plasticity, cancer cells can invade the ECM through a non-protease-dependent manner, meaning cells can squeeze through collagen networks in an ameboid-like phenotype (28, 105). However, research has demonstrated that HT-1080 or MDA-MB-231 cells with silenced MT1-MMP are unable to adopt an invasive ameboid-like phenotype when embedded in three-dimensional type I collagen gels. This finding indicates a critical requirement for membrane-anchored MT1-MMP in the process of cancer cell invasion (106). Although this conclusion contradicts some findings from other studies, the recombinant collagen gel structures used in those studies had defects (107).

### 3.3 Tumor immune suppression

**Figure 4:**
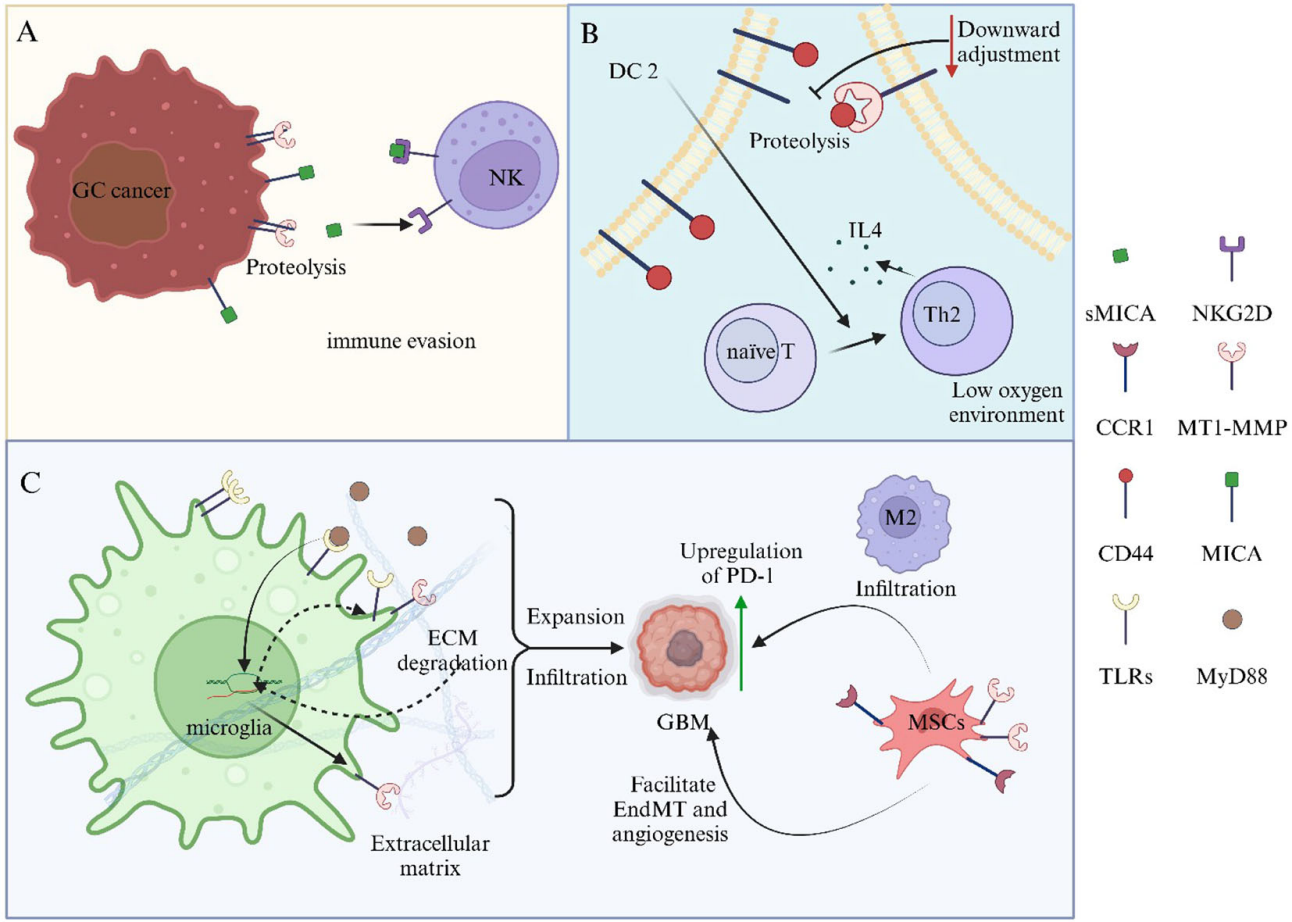
MT1-MMP plays a crucial role in regulating the immune microenvironment within the tumor microenvironment. **(A)** MT1-MMP influences the phenotypic differentiation of dendritic cells, which in turn modulates T cell phenotypes and cytokine secretion. **(B)** MT1-MMP downregulates surface molecules on tumor cells, thereby inhibiting NK cell activation and facilitating tumor immune evasion. **(C)** MT1-MMP impacts microglia and mesenchymal stem cells, contributing to immune suppression, enhancing tumor cell proliferation, angiogenesis, and invasive infiltration.

MT1-MMP exhibits a strong correlation with tumor immune invasion and significantly influences the onset and progression of various types of tumors (108). MT1-MMP is translocated intracellularly to the nucleus, where it modulates the induction of macrophage immune responses by promoting the expression and activation of the PI3Kδ/Akt/GSK3β signaling pathway (109). In the hypoxic tumor microenvironment, the downregulation of KIF2A leads to a reduction in the membrane surface expression of MT1-MMP. This, in turn, decreases the shedding of CD44 in dendritic cells, which induces a DC2-like phenotype and promotes the Th2 polarization of naïve T cells, resulting in an increased production of interleukin-4 (110) (**Figure 4A**). Recent studies have identified a novel mechanism by which the upregulation of PLEK2 in gastric cancer cells facilitates evasion from natural killer cell cytotoxicity. Specifically, PLEK2 enhances the expression of MT1-MMP via the PI3K-AKT-Sp1 signaling pathway. This process results in the shedding of MICA, which subsequently inhibits the activation of NKG2D on NK cells, thereby promoting immune evasion (111, 112) (**Figure 4B**). In GBM, the Toll-like receptor 2 signaling pathway influences microglia, specifically tumor-associated microglia, leading to an upregulation of MT1-MMP gene and protein expression, which subsequently facilitates tumor progression (113). Additionally, myeloid cells expressing MT1-MMP mediate immune suppression via chemokine receptor 1(CCR1), with CCR1 upregulation associated with M2 macrophage infiltration and increased PD-L1 expression (91) (**Figure 4C**).

### 3.4 Tumor cell proliferation

During tumorigenesis and development, MT1-MMP also participates in the process of tumor cell proliferation. Relevant literature reports that the growth and survival of melanoma require activated Notch1, which is generally considered to be related to the proteolytic activity of ADAM10 and ADAM17. Jun Ma et al. identified a significant association between active Notch1 and MT1-MMP in melanoma. This correlation was not observed with ADAM10 and ADAM17, thereby elucidating a novel mechanism through which MT1-MMP activates Notch1 in melanoma. This indicates that MT1-MMP-dependent activation of Notch1 promotes melanoma cell growth (114). Furthermore, in the context of gastric cancer, the downregulation of MT1-MMP expression significantly impedes cell proliferation and invasion by modulating the expression levels of vimentin and E-cadherin (115) The ERK1/2 and PI3K/AKT signaling pathways, which are regulated by MT1-MMP in MSCs, play a crucial role in modulating invasiveness and proliferative capacity (116). Additionally, the process of EMT is observed during tumor progression, facilitating the acquisition of a mesenchymal phenotype. We have reason to believe that MT1-MMP-dependent cell proliferation in MSC may also occur in tumor cells that have acquired a mesenchymal phenotype.

## 4 Current status and prospects of MT1-MMP targeted antitumor therapies

Despite the critical biological roles of MT1-MMP in tumorigenesis and progression, coupled with its distinctive structural characteristics that render it a promising target, the current scarcity of research data pertaining to cell therapy, along with an inadequate comprehension of the multifaceted functions of MT1-MMP, indicates that its substantial potential for application in cell therapy necessitates further extensive investigation over an extended period. In mouse models, deficiency of MT1-MMP results in delayed bone development, impaired angiogenesis, severe fibrosis, arthritis, connective tissue diseases, and premature death (117). Research has demonstrated that cartilage-specific, tamoxifen-induced MT1-MMP knockout mice exhibit significant chondrocyte hypertrophy; however, they do not display synovial hyperplasia or evident arthritis (118). This indicates that the potential therapeutic application of targeting MT1-MMP necessitates highly cell type-specific inhibition.

The study conducted by Ragusa et al. found that the absence of PROX1 in mouse intestinal tumors leads to the overactivation of MT1-MMP, resulting in tumors that grow slowly but exhibit increased aggressiveness and promote connective tissue proliferation. Subsequent research revealed that tumors with overactivated MT1-MMP displayed a denser and more complex microvascular network, reduced immune cell infiltration, and resistance to chemotherapy. Furthermore, organoid transplant tumors with PROX1-induced overactivation of MT1-MMP also demonstrated slow tumor growth, increased stromal content, and elevated levels of TGF-β, Ctgf, Opn, and IL-1β (20, 119). Notably, in breast cancer, MT1-MMP expression is heightened in chemotherapy-responsive cancer cells and stromal cells (120). The variations in MT1-MMP expression and chemotherapy resistance across different tumors suggest that elucidating the detailed mechanisms of MT1-MMP in various physiological and pathological processes will be essential for the development of targeted anti-tumor therapies aimed at MT1-MMP.

### 4.1 Current antitumor therapies targeting MT1-MMP

Despite the established pathological roles of MMPs in a variety of diseases and their potential as therapeutic targets, over 50 MMP inhibitors have failed to demonstrate efficacy in clinical trials for various cancer types during the initial phases of drug development (121). It is noteworthy that the failures of these clinical trials were caused by multiple factors (122–124), including a lack of specificity in the drugs themselves and insufficient understanding of the biological complexity of MMPs in diseases, which should receive more attention. Various strategies have been implemented to improve the targeting of MMPs inhibitor (MMPI). These strategies include, but are not limited to, the utilization of protein engineering to generate specific antibodies, the creation of targeted delivery vehicles, and the application of click chemistry techniques (125, 126). The initial development of MMPI primarily focused on broad-spectrum agents such as Rebimastat, which aimed to target multiple MMPs. However, Rebimastat failed in clinical trials (127–129). The membrane-anchored structure and critical role of MT1-MMP in tumorigenesis have led to increased interest in the development of specific antibodies targeting MT1-MMP, resulting in notable advancements in this area.

Research has reported that the MT1-MMP antibody Fab3369 remodels the tumor immune microenvironment and inhibits lung metastasis in the MDA-MB-231 triple-negative breast cancer mouse model (130). In the 4T1 mouse model, the MT1-MMP antibody DX-2400 promotes M1-like phenotype differentiation, inhibits TGF-β activation, and suppresses tumor growth (120). In addition, the targeted drug MC-T-DOX, developed based on the proteolytic activity of MT1-MMP and its enriched expression characteristics in the tumor microenvironment, utilizes an MT1-MMP-specific cleavable RGD-mimetic cyclic peptide to deliver liposomes loaded with doxorubicin (DOX). This approach integrates the promotion of tumor vascular regulation with intelligent nanodrug delivery, thereby enhancing the treatment efficacy for pancreatic cancer (5). 99mTc-(HYNIC-AF7p) is a 99mTc-labeled MT1-MMP specific binding peptide, which shows great potential in *in vivo* MT1MMP targeting detection and may become a promising molecular imaging probe to aid in early diagnosis of breast cancer (131). ICT3205 is a peptide-based prodrug conjugate that consists of a specific peptide targeting MT1-MMP and paclitaxel. This conjugate demonstrates improved pharmacokinetics and efficacy in targeting prostate cancer for the delivery of active paclitaxel (132). BT1769 is a bicyclic peptide designed to target the tumor antigen MT1-MMP. It is linked to the cytotoxic agent monomethyl auristatin E through a molecular spacer and a cleavable linker, resulting in a significant inhibition of tumor growth in patient-derived xenograft models of osteosarcoma (133, 134). ND-322 is characterized as a slowly binding inhibitor of MT1-MMP and MMP2, demonstrating the capacity to impede the growth, migration, and invasion of various melanoma cell lines. Furthermore, it has been shown to significantly diminish tumor growth and metastasis in an *in situ* mouse model of melanoma (135). Pb-BCY20603 is a radioactive conjugate drug designed to target MT1-MMP. Experimental results obtained from mouse models suggest that the bicyclic peptide targeting MT1-MMP can serve as a high-contrast imaging probe for clinical diagnosis and demonstrates significant potential for application in targeted therapy (136). BT1718 is a bicyclic drug conjugate that contains a constrained bicyclic peptide (Bicycle^®^), which binds to MT1-MMP with high affinity and specificity, covalently linked to the potent microtubule inhibitor DM1 via a sterically hindered disulfide bond. In clinical trial, the first phase stabilized tumors in 54% of candidate patients and showed good tolerability, currently traversing phase 2 expansion trials (137–139).

**Figure 5:**
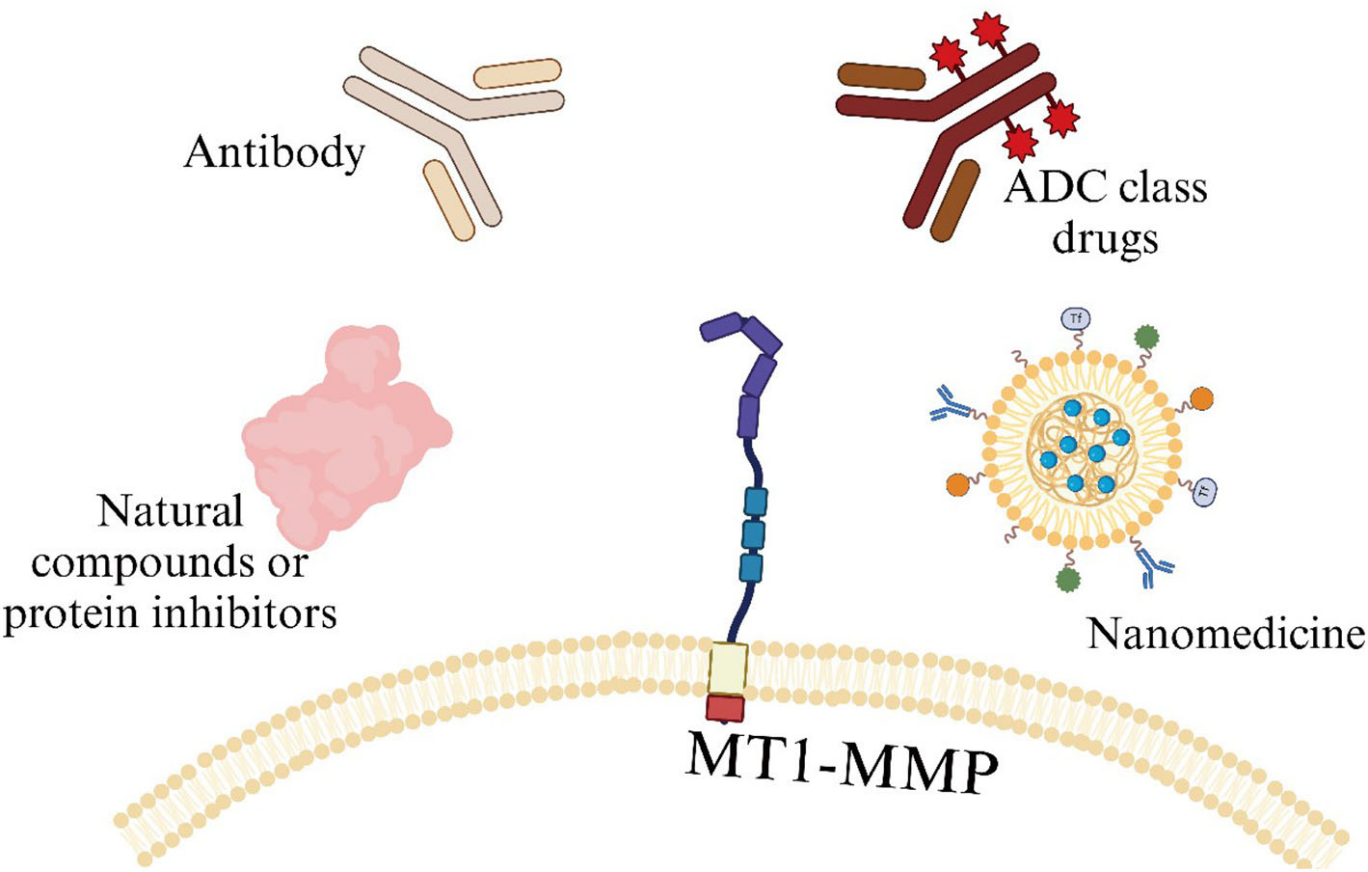
Schematic diagram of various molecules currently targeting MT1-MMP in TME.

Surprisingly, despite the good anti-tumor effects demonstrated by the aforementioned MT1-MMP targeting studies in the fields of small molecule inhibitors and targeted drugs (**Table 1**) (**Figure 5**), research related to targeting MT1-MMP in cell therapy is quite scarce.

**Table 1:** Research progress of drugs targeting MT1-MMP.

Drug	Drug Type	Target	Active Indication	Drug Highest Phase
BT-1718 (137–139)	Peptide drug conjugates	MMP14 × Tubulin	Neoplasms Advanced Malignant Solid Neoplasm Non-Small Cell Lung Cancer	Phase 2
BT-1769 (133, 134)	Peptide drug conjugates	MMP14x Tubulin	Osteosarcomas	Preclinical
212Pb BCY- 20603 (136)	Small molecule drug Diagnostic radiopharmaceuticals	MMP14	Neoplasms	Preclinical
Pb-BCY20603 (136)	Peptide Conjugate Radionuclide Therapeutic radiopharmaceuticals	MMP14	Neoplasms	Preclinical
ICT3205 (132)	Peptide drug conjugates	MMP14	Neoplasms Prostatic Cancer	Preclinical
Rebimastat (127–129)	Small molecule drug	MMP1	Non-Small Cell	Discontinued (Phase 3)
		MMP2	Lung Cancer	
		MMP8	Kaposi Sarcoma	
		MMP9	Prostatic Cancer	
		MMP14	Breast Cancer	
KD-014 (120)	Monoclonal antibody	MMP14	Solid tumor	Pending (Discovery)
ND-322 (135)	Small molecule drug	MMP14 MMP2	Melanoma	Pending (Discovery)

### 4.2 Prospects and challenges of targeting MT1-MMP for anti-tumor therapy

#### 4.2.1 MT1-MMP inhibitors

The biological functions of MT1-MMP in tumor cells and its regulatory processes are indeed complex and variable. As the understanding of the diverse functions of MT1-MMP in tumors becomes more comprehensive, its potential as a target for anti-tumor therapies is increasingly recognized. Nevertheless, the prior shortcomings observed in MMPI clinical trials necessitate a more thorough investigation into the diverse roles of MT1-MMP across various tumor types, at different temporal stages, and under distinct physiological conditions (140). Indeed, MT1-MMP may exert contrasting effects on fundamental aspects of tumor progression depending on varying conditions (121, 141). This variability presents considerable challenges for the development of anti-tumor pharmacological agents that target MT1-MMP. Specific inhibitors designed to target MT1-MMP and inhibit particular functions have been validated through preclinical experiments (142). This approach can specifically limit some activities of MT1-MMP and reduce the drug’s toxic side effects, but it also somewhat weakens the anti-tumor effect and raises higher demands for assessing disease progression during drug use. Furthermore, the development of carriers for drug delivery that specifically target tumor-associated MT1-MMP may enhance the penetration of therapeutic agents into solid tumors. However, existing studies have indicated that the *in vivo* efficacy of these carriers is not as optimal as anticipated (143, 144). Considering that tumors represent complex systemic diseases, the effects attainable through partial functional inhibition aimed at a single target are inherently limited. Consequently, the integration of this approach with other anti-tumor therapies is anticipated to be a prevailing trend.

#### 4.2.2 MT1-MMP targeted drugs

Antibody-drug conjugates (ADCs) have achieved great success in recent years in the field of targeted anti-tumor drugs. Currently approved ADCs typically target antigens that are overexpressed in cancer cells (145). Following ADCs, PDCs present several advantages, including enhanced tumor penetration, reduced immunogenicity, and decreased production costs. PDC drugs targeting MT1MMP have already demonstrated excellent anti-tumor effects in preclinical studies (133, 137, 138).

Furthermore, BRC drugs have demonstrated promise in clinical diagnosis and targeted therapy (136). This approach effectively circumvents a comprehensive investigation into the intricate biological roles of MT1-MMP in tumors by administering small molecule toxins or radioactive conjugates that specifically target antigens overexpressed in neoplastic tissues. However, a one-size-fits-all antitumor approach also brings new issues, as MT1-MMP, while overexpressed in tumors and their stromal cells, also has low-level expression in normal tissue cells. Furthermore, due to immune evasion mechanisms such as antigen modulation, antigen masking and covering, and antigen exhaustion present in tumor cells (146–148). Recent advancements in ADC anti-tumor therapies have enhanced tumor targeting through modified antibodies, multispecific antibodies, and conditionally activated antibody prodrug conjugates (149, 150). These developments offer valuable insights for the creation and refinement of targeted drugs.

The internalization efficiency of targeted antibodies in ADCs significantly influences the overall efficacy of these therapies, and this internalization is typically antibody-dependent (151). The previous section described a mechanism for regulating the activity of membrane surface MT1-MMP that relies on the endocytosis and transport of MT1-MMP, suggesting that the development of targeted drugs against MT1-MMP may enhance internalization efficiency. Although ADCs demonstrate considerable efficacy, they are still associated with notable side effects during clinical application, including hematological adverse reactions, neurotoxicity, and hepatotoxicity (152). It is crucial to recognize that off-target toxicity resulting from small molecule toxins is a major contributor to adverse reactions in ADCs (153, 154). This underscores the necessity of further improving drug stability and enhancing targeted delivery efficiency in the ongoing development of MT1-MMP-targeted ADCs to mitigate the occurrence of adverse reactions.

#### 4.2.3 Application of MT1-MMP in cell therapy

In the field of anti-tumor therapy, cell therapy has achieved remarkable results, especially in Chimeric antigen receptor (CAR)-T cell therapy for hematological tumors (155). However, there is currently a lack of cell therapies targeting MT1-MMP. Some studies have shown that CAR-147 macrophage therapy with overexpression of activated CD147 can degrade ECM by increasing the expression of MMPs, thereby promoting T cell infiltration and inhibiting tumor growth (156). Although MT1-MMP is involved in this process, it has not yet been the primary research focus in cell therapy.

**Figure 6:**
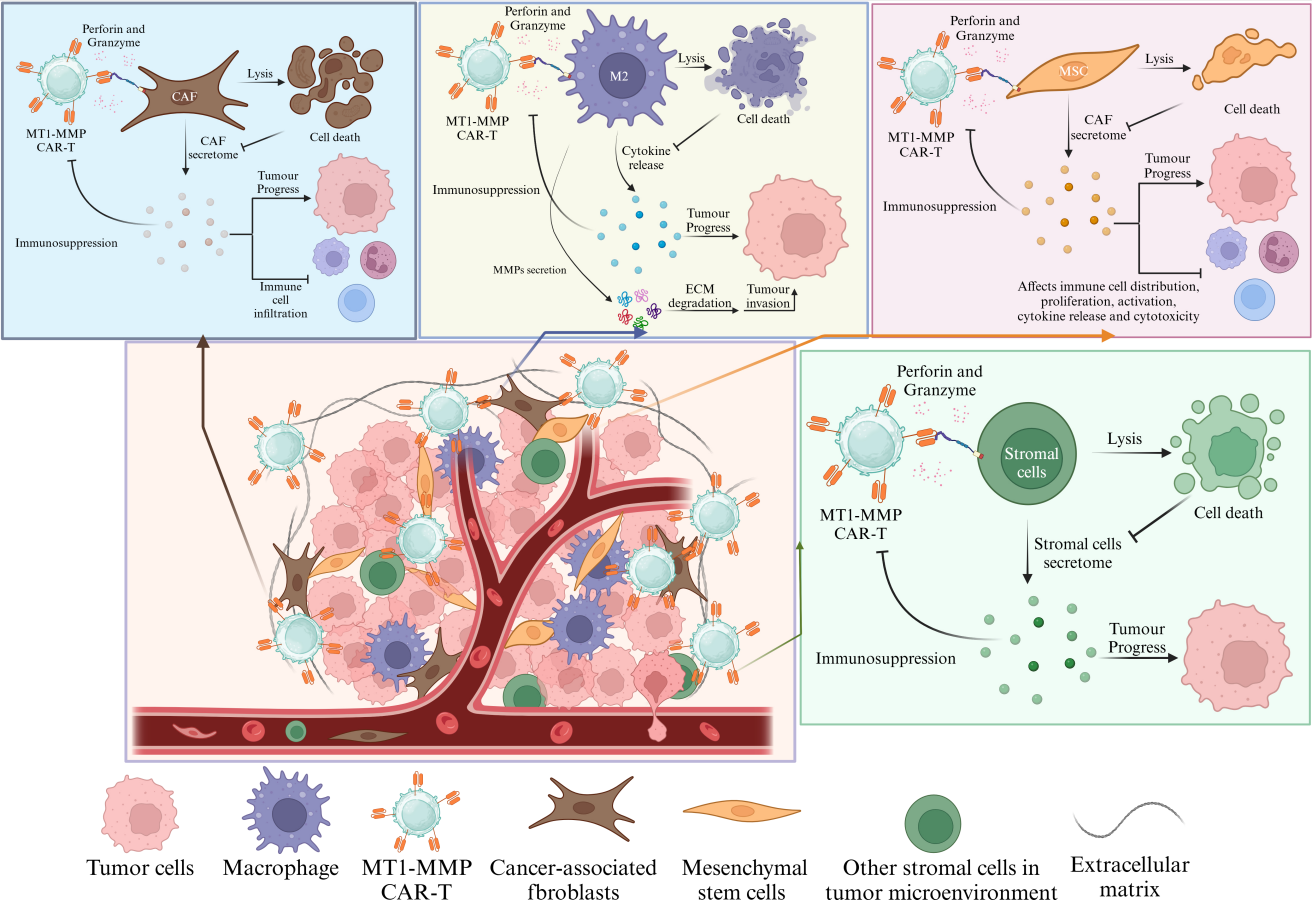
CAR-T cells that specifically target MT1-MMP are capable of inducing cytotoxic effects on both tumor cells and tumor-associated stromal cells that express the MT1-MMP antigen. MT1-MMP is predominantly found in tumor cells and a range of immunosuppressive stromal cell populations. The targeted lysis of MT1-MMP-positive stromal cells mitigates the release of various cytokines and dismantles physical barriers, thereby facilitating the infiltration of immune cells, reducing immune suppression within the tumor microenvironment, and ultimately inhibiting tumor progression.

In this section, we investigate the potential application of MT1-MMP in CAR-T cell therapy. The TME associated with solid tumors considerably restricts the effectiveness of CAR-T cell therapy. In the TME, CAR-T cells must navigate through the tumor ECM and withstand various environmental challenges, including immune suppression, hypoxia, and low pH, to effectively target and engage tumor cells (157–159). Although numerous strategies have been proposed to mitigate the limitations of the tumor microenvironment, the identification of appropriate targets remains essential for the advancement of CAR-T research. Notably, almost all currently studied solid tumor CAR-T target antigens can be classified into four categories: tumor-specific antigens(TSA), tumor-associated antigens(TAA), cancer-associated stromal cell surface antigens(CASC), and glycosphingolipid antigens (160). Research indicates that CAR-T cells targeting fibroblast activation protein (FAP), when combined with CAR-T cells targeting claudin 18.2, can effectively eliminate tumor-associated fibroblasts in the treatment of pancreatic cancer. This combination promotes the infiltration of claudin 18.2-targeted CAR-T cells and reduces immune suppression within the TME, thereby enhancing the anti-solid tumor effects of CAR-T therapy (161). Similar efficacy has been observed with the combination of FAP-targeted CAR-T cells and glypican-3 (GPC3)-targeted CAR-T cells in the treatment of liver cancer (162). These studies suggest that the concurrent use of CAR-T cells targeting CASC alongside those targeting tumor-associated antigens TAA may represent an effective strategy for CAR-T therapy to overcome solid tumors. Numerous studies have demonstrated that MT1-MMP functions as a tumor-associated antigen and is overexpressed on the surface of various cancer cells, as well as their cancer-associated stromal cells (6, 108, 163). It is plausible to hypothesize that CAR-T cells directed against MT1-MMP may effectively eliminate cells that express MT1-MMP. Given that MT1-MMP is expressed in specific tumor cells as well as in cancer associated fibroblasts, macrophages, epithelial cells, and bone marrow-derived MSCs (164, 165), CAR-T cells that target MT1-MMP may exhibit enhanced efficacy in the elimination of solid tumors. This efficacy may stem not only from immune infiltration resulting from the destruction of stromal cells but also from a reduction in immune suppression through the elimination of immunosuppressive cells, in addition to the direct targeting of tumor cells that express MT1-MMP (**Figure 6**).

In addition, a major challenge faced by CAR-T therapy is that tumor cells can achieve immune evasion by downregulating surface antigens, which is also considered a primary reason for tumor recurrence after CAR-T treatment (166, 167). As previously discussed, the expression of MT1-MMP is closely linked to processes such as angiogenesis, tumor invasion, and immune suppression during tumor progression. Reducing the expression of MT1-MMP may enhance the efficacy of other antitumor immunotherapies, thereby making the combination of MT1-MMP-targeted CAR-T therapy with additional anti-tumor immunotherapies a viable option. Although MT1-MMP is expressed at low levels in normal tissues and produces soluble functional fragments, these characteristics do not preclude its potential as a target for CAR-T cell therapy. As a matter of fact, the results from clinical phase I trials for BT1718 indicate that direct cell killing targeting MT1-MMP has acceptable side effects at appropriate doses. Furthermore, off-target effects, commonly referred to as on-target off tumor effects, have become a notable issue within the framework of CAR-T cell therapy. Numerous strategies have been suggested to alleviate CAR-T OTOT, including the modification of the affinity of targeting sequences, the implementation of logic-gated CAR-T systems, and the incorporation of exogenous control structures (168). CAR-T cells recognize and eliminate target cells, releasing a substantial amount of cytokines and further activating immune cells. This process creates a positive feedback loop of cytokine release, resulting in a systemic inflammatory response and significant damage to tissues and organs, a condition known as cytokine release syndrome (CRS). CRS is the most common and notable acute toxic reaction associated with CAR-T therapy in the treatment of hematological malignancies (169). Currently, in addition to optimizing the structure of CAR-T cells to reduce the incidence of CRS, the FDA has approved a range of clinical treatment options for CRS, including the IL-6 receptor blocker tocilizumab and the TNF-α blocker etanercept (170).

Excitingly, Roei D and colleagues have identified the presence of endogenous natural antibodies against MT1-MMP in the tumor tissues of high-grade serous ovarian cancer. These antibodies have the capacity to specifically target tumor cells and bind to tumor-associated molecules (171).

Unfortunately, it has not yet been proven that these endogenous MT1-MMP antibodies are specific (171). The generation of these antibodies is unexpected and introduces renewed potential into the field of cancer immunotherapy. It is anticipated that this endogenous and tumor-specific targeting antibody, following thorough validation and optimization, could evolve into an almost CAR-T targeting sequence. It is noteworthy that a specific type of regulatory activated CAR-T therapy, which leverages the enrichment of MMPs within the TME, has the potential to enhance the safety profile of CAR-T treatment while simultaneously improving anti-tumor efficacy (172). We posit that comparable effects may be attained through the indirect targeting of MT1-MMP-optimized CAR-T cells.

## 5 Summary

In this review, we primarily summarize the various physiological and pathological aspects involved in the role of MT1-MMP in tumorigenesis and development. We also outline the current progress of anti-tumor therapies targeting MT1-MMP. Finally, we discuss the potential and limitations of targeting MT1-MMP for anti-tumor purposes. For a long time, the TME has been a significant factor contributing to the poor efficacy of many anti-tumor therapies. Most anti-tumor treatments typically target only specific components of the tumor as a systemic disease. While this approach can somewhat limit tumor progression, the inherent complexity of tumors makes it challenging for a single treatment strategy to achieve favorable therapeutic outcomes. Although numerous studies have demonstrated that combination therapies can yield enhanced anti-tumor effects, investigating whether the combined use of different therapies can achieve significant synergistic effects requires considerable time and effort. Here, we propose a novel target, MT1-MMP, which is abundantly expressed in both tumors and the stromal cells of their microenvironment. This characteristic endows certain anti-tumor therapies targeting MT1-MMP with the potential to view tumors and the TME as an integrated system, representing a new direction for overcoming the challenges of target selection in solid tumors. It is crucial to emphasize that the development of anti-tumor therapies targeting MT1-MMP is still in the exploratory stage, and extensive research is needed to uncover the specific effects of these therapies.
